# Impaired immune signaling and changes in the lung microbiome precede secondary bacterial pneumonia in COVID-19

**DOI:** 10.21203/rs.3.rs-380803/v1

**Published:** 2021-04-23

**Authors:** Alexandra Tsitsiklis, Beth Shoshana Zha, Ashley Byrne, Catherine Devoe, Sophia Levan, Elze Rackaityte, Sara Sunshine, Eran Mick, Rajani Ghale, Alejandra Jauregui, Aartik Sarma, Norma Neff, Paula Hayakawa Serpa, Thomas J. Deiss, Amy Kistler, Sidney Carrillo, K. Mark Ansel, Aleksandra Leligdowicz, Stephanie Christenson, Norman Jones, Bing Wu, Spyros Darmanis, Michael A. Matthay, Susan V. Lynch, Joseph L. DeRisi, COMET Consortium, Carolyn M. Hendrickson, Kirsten N. Kangelaris, Matthew F. Krummel, Prescott G. Woodruff, David J. Erle, Oren Rosenberg, Carolyn S. Calfee, Charles R. Langelier

**Affiliations:** 1 Department of Medicine, Division of Infectious Diseases, University of California San Francisco, San Francisco, CA, USA; 2 Department of Medicine, Division of Pulmonary, Critical Care, Allergy and Sleep Medicine, University of California San Francisco, San Francisco, CA, USA; 3 Chan Zuckerberg Biohub, San Francisco, CA, USA; 4 Department of Medicine, University of California San Francisco, San Francisco, CA, USA; 5 Department of Biochemistry and Biophysics, University of California San Francisco, San Francisco, CA, USA; 6 Department of Microbiology and Immunology, University of California, San Francisco, CA, USA; 7 Sandler Asthma Basic Research Center, University of California, San Francisco, CA, USA; 8 Department of Experimental Medicine, University of California, San Francisco, CA, USA; 9 Genentech, Inc. San Francisco, CA, USA.; 10 Department of Gastroenterology, University of California, San Francisco, CA, USA; 11 Benioff Center for Microbiome Medicine, University of California, San Francisco, CA, USA; 12 Department of Pathology, University of California, San Francisco, CA, USA; 13 Lung Biology Center, University of California, San Francisco, CA, USA; 14 UCSF CoLabs, University of California, San Francisco, CA, USA

**Keywords:** COVID-19, SARS-CoV-2, secondary bacterial pneumonia, VAP, metagenomics, scRNA-seq

## Abstract

Secondary bacterial infections, including ventilator-associated pneumonia (VAP), lead to worse clinical outcomes and increased mortality following viral respiratory infections including in patients with coronavirus disease 2019 (COVID-19). Using a combination of tracheal aspirate bulk and single-cell RNA sequencing (scRNA-seq) we assessed lower respiratory tract immune responses and microbiome dynamics in 28 COVID-19 patients, 15 of whom developed VAP, and eight critically ill uninfected controls. Two days before VAP onset we observed a transcriptional signature of bacterial infection. Two weeks prior to VAP onset, following intubation, we observed a striking impairment in immune signaling in COVID-19 patients who developed VAP. Longitudinal metatranscriptomic analysis revealed disruption of lung microbiome community composition in patients with VAP, providing a connection between dysregulated immune signaling and outgrowth of opportunistic pathogens. These findings suggest that COVID-19 patients who develop VAP have impaired antibacterial immune defense detectable weeks before secondary infection onset.

## Introduction

Secondary bacterial pneumonia results in significant morbidity and mortality in patients with viral lower respiratory tract infections (LRTI)^1^. This problem was evident in the 1918 influenza pandemic during which the majority of deaths were ultimately attributed to secondary bacterial pneumonia^2^. SARS-CoV-2 infection, like influenza, confers an increased risk of late onset secondary bacterial infection, often manifesting as ventilator-associated pneumonia (VAP)^3^. Marked heterogeneity exists with respect to the risk of VAP in patients with coronavirus disease 2019 (COVID-19), with incidence ranging from 12–87% between published cohort studies^4–7^.

The mechanisms underlying VAP susceptibility in COVID-19 remain unknown, and no biomarkers yet exist to inform risk of VAP at the time of intubation. Animal models of influenza may provide some insight, suggesting a role for interferon-mediated suppression of cytokines essential for bacterial defense, including neutrophil recruitment, antimicrobial peptide production and the Th17 response^8–10^. Few human immunoprofiling studies have been conducted in VAP however, and none have been reported in a prospective cohort of COVID-19 patients.

Lower respiratory infections represent a dynamic relationship between pathogen, host response and the lung microbiome^11^. Despite their interconnected roles, no studies to date have simultaneously profiled host immune responses and lung microbiome dynamics in the context of VAP. For instance, while prior work has described lung microbiome disruption in patients with VAP^11,12^, the question of whether host immune responses following viral infection may contribute to this dysbiosis, leading to subsequent infection, remains unanswered.

Given the marked heterogeneity in VAP incidence among patients with COVID-19^4–7^, as well as gaps in mechanistic understanding of secondary bacterial pneumonia, we sought to assess the molecular determinants of VAP in the setting of SARS-CoV-2 infection. We employed a systems biology approach involving immunoprofiling the host transcriptional response and simultaneously assessing lung microbiome dynamics, using a combination of bulk and single cell RNA sequencing and extensive clinical phenotyping. We observed a striking impairment in antibacterial immune signaling at the time of intubation, that correlated with disruption of the lung microbiome, weeks before the onset of VAP.

## Results

**Figure 1:**
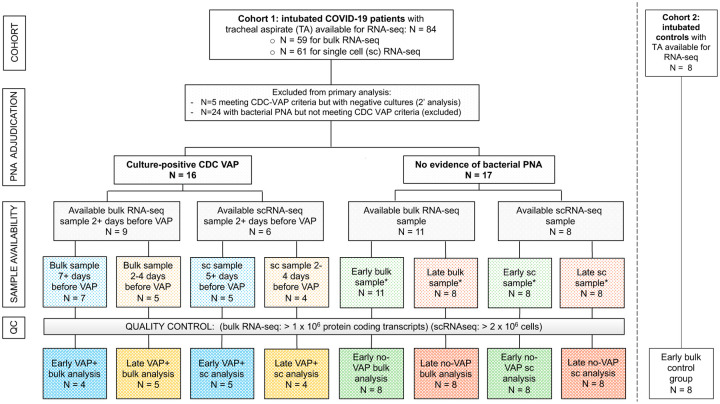
Study flowchart. Two patient cohorts were studied. Cohort 1 consisted of COVID-19 patients from the COVID Multiphenotyping for Effective Therapies (COMET) / Immunophenotyping Assessment in a COVID-19 Cohort (IMPACC) studies (described in Methods). Cohort 2 consisted of critically ill intubated control patients from a prior prospective cohort study led by our research group^18^. The “early” samples were the first available tracheal aspirate specimens after intubation. For COVID-19 patients who developed VAP, the “late” samples were obtained a median of two days before VAP onset. Timing of sample collection with respect to VAP versus No-VAP groups was matched at “early” and “late” time points. Controls included eight critically ill, mechanically ventilated patients without LRTI. All COVID-19 patients included in the primary bulk analysis were also included in the longitudinal host expression and microbiome analyses. Abbreviations: VAP=ventilator-associated pneumonia; TA=tracheal aspirate; QC=quality control; sc or scRNA-seq= single cell RNA sequencing; PNA=pneumonia; CDC=United States Centers for Disease Control and Prevention.

We conducted a prospective case-control study of adults requiring mechanical ventilation for COVID-19 or for illnesses other than pneumonia. Of 84 patients with COVID-19 initially enrolled, tracheal aspirate (TA) specimens from 28 patients met inclusion criteria for analysis (Methods, Figure 1). In addition, eight critically ill patients from a second cohort (Study 2, Methods) were included as controls. Patients were enrolled at one tertiary care hospital and one safety net hospital in San Francisco, California under research protocols approved by the University of California San Francisco Institutional Review Board (Methods). We collected TA periodically following intubation and performed bulk and scRNA-seq (Methods).

Patients with VAP were adjudicated using the United States Centers for Disease Control (CDC) definition^13^, including a requirement for a positive bacterial TA culture (N=10). Patients who met CDC VAP criteria but had negative bacterial TA cultures were only included in a secondary analysis (N=5). We defined onset of VAP as the first day a patient developed any of the criteria used to meet the definition, in accordance with CDC guidance. Patients who did not meet the CDC-NHSN criteria for VAP, and for whom there was no sustained clinical suspicion for bacterial pneumonia during the admission, were adjudicated as No-VAP (N=13).

We compared lower respiratory tract host transcriptional responses between the VAP and No-VAP groups at two time points. “Early” time point TA samples were collected a median of two days post-intubation and 17 days before VAP onset (bulk RNA-seq analysis) or nine days before VAP onset (scRNA-seq). “Late” time point samples were collected a median of two days before VAP onset for both bulk and scRNA-seq analyses and compared against samples collected from No-VAP patients at similar timepoints post-intubation (Figure 1, Table S1, Table S2). We additionally evaluated eight intubated patients with non-pneumonia illnesses as controls at the “early” time-point. There were no significant differences between groups with respect to age, gender, race or ethnicity (Table S1, S2). In addition, there were no differences between groups with respect to in-hospital receipt of any immunosuppressant or antibiotics prior to sample collection (Table S3).

### COVID-19 VAP is associated with a transcriptional signature of bacterial infection two days before VAP onset

**Figure 2:**
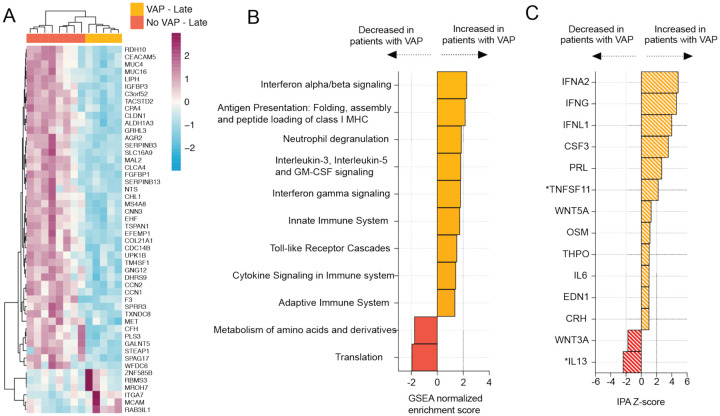
COVID-19 VAP is associated with a lower respiratory tract transcriptional signature of bacterial infection 2 days before VAP onset. **A)** Heatmap of the top 50 differentially expressed genes by adjusted P-value between COVID-19 patients who developed VAP (yellow) versus those who did not (red) at the “late” time-point, 2 days before the onset of VAP, from bulk RNA-seq. **B)** Gene set enrichment analysis (GSEA) at the “late” time-point based on differential gene expression analyses. GSEA results were considered significant with an adjusted P-value <0.05. **C)** Ingenuity Pathway Analysis (IPA) of upstream cytokines at the “late” time-point based on differential gene expression analyses. IPA results were considered significant with a Z-score absolute value >2 and overlap P-value <0.05. *Denotes cytokines with an overlap P-value < 0.1. All pathways and cytokines are shown in Supplementary data files 2 and 3.

We began by assessing the lower respiratory host transcriptional response two days preceding VAP onset in COVID-19 patients. Differential gene expression analysis was carried out on TA bulk RNA-seq data from five patients who developed VAP (samples collected a median of two days before VAP onset) and eight patients who did not develop VAP collected within a similar time frame after intubation (Table S1). We identified 436 differentially expressed genes at a False Discovery Rate (FDR) < 0.1 (Figure 2A) and performed gene set enrichment analysis (GSEA) (Figure 2B). The patients who developed VAP exhibited upregulation of pathways related to anti-bacterial immune responses, such as neutrophil degranulation, toll-like receptor signaling, cytokine signaling, and antigen presentation (Figure 2B). Interferon alpha/beta signaling was the most upregulated pathway, suggesting prolonged viral infection in patients with VAP. Ingenuity pathway analysis (IPA) additionally predicted broad activation of upstream inflammatory cytokines in patients who developed VAP, in particular IFNα and IFNγ (Figure 2C).

### COVID-19 patients who develop VAP have attenuated immune signaling two weeks before VAP onset

**Figure 3:**
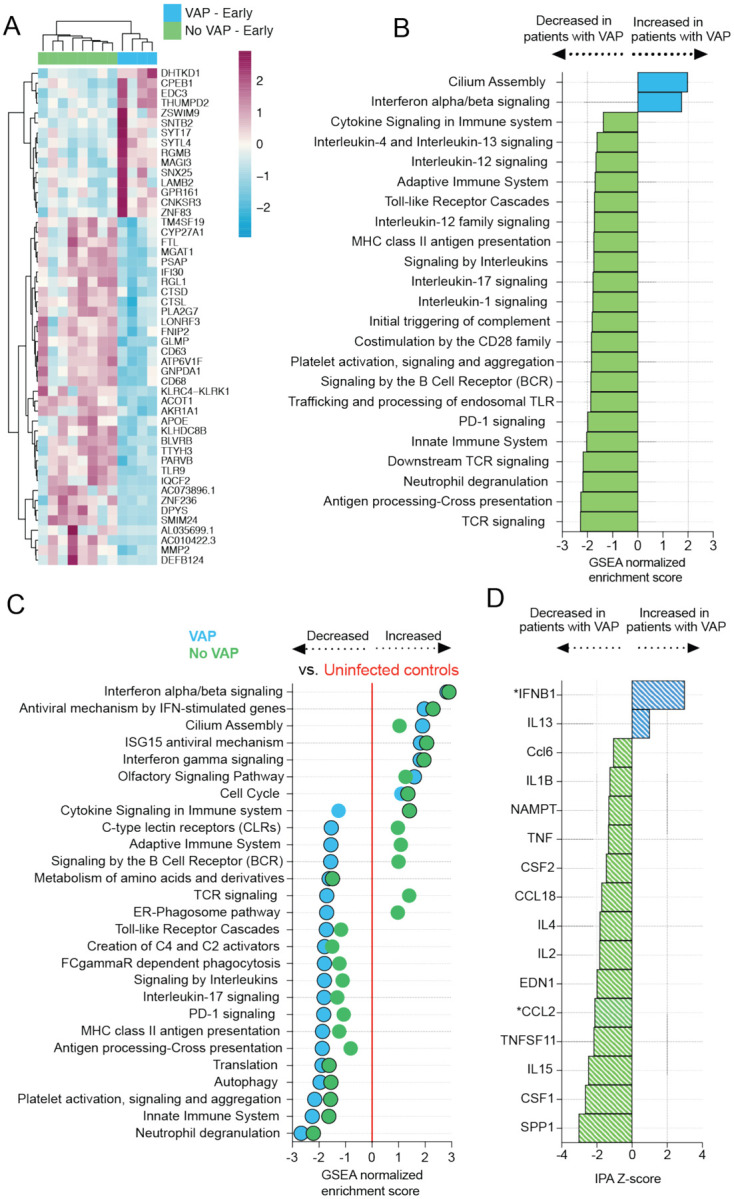
COVID-19 patients who develop VAP have attenuated immune signaling in the lower respiratory tract two weeks before onset of secondary bacterial pneumonia. **A)** Heatmap of the top 50 differentially expressed genes by adjusted P-value between COVID-19 patients who developed VAP (blue) versus those who did not (green) at the “early” time-point from bulk RNA-seq. **B)** Gene set enrichment analysis at the “early” time-point based on differential gene expression analyses. GSEA results were considered significant with an adjusted P-value <0.05. **C)** Expression of GSEA pathways at the “early” time-point with respect to a baseline of uninfected, intubated controls. Pathways were selected from the GSEA results if they had an adjusted P-value <0.05 in at least one of the comparisons (VAP vs controls or No-VAP vs controls). Pathways with an adjusted P-value <0.05 when compared to controls are indicated by circles with a black outline. **D)** Ingenuity Pathway Analysis (IPA) of upstream cytokines at the “early” time-point based on differential gene expression analyses. IPA results were considered significant with a Z-score absolute value >2 and overlap P-value <0.05. *Denotes cytokines with an overlap P-value <0.1. All pathways and cytokines are shown in Supplementary data files 2 and 3.

Given our findings of a unique lower respiratory host transcriptional signature in the 48 hours preceding VAP onset, we next asked whether differences in host immune signaling might exist even earlier, two or more weeks before clinical diagnosis of VAP, and whether such differences might explain the increased susceptibility to secondary bacterial infection in these patients. We thus compared TA gene expression soon after the time of intubation between patients who eventually developed VAP (samples collected a median of two days post-intubation, 17 days before VAP onset, n=4) and patients who did not develop VAP (samples collected a median of two days after intubation, n = 8) (Table 1). We identified 154 differentially expressed genes at FDR <0.1. The COVID-19 patients who developed VAP had lower expression of several genes with roles in innate immunity including *IFI30, MMP2, TLR9*, and *DEFB124* (Figure 3A). GSEA further revealed that patients who developed VAP had lower expression of pathways related to antibacterial immune responses including neutrophil degranulation, toll-like receptor signaling, IL-17 signaling, antigen presentation and complement pathways and higher expression of IFN-alpha/beta signaling pathways, more than two weeks before the onset of VAP (Figure 3B). Additionally, pathways related to adaptive immunity such as T and B cell receptor signaling were also downregulated in patients who subsequently developed VAP (Figure 3B).

To gauge the degree of immune signaling suppression compared to controls, we performed a similar analysis on critically ill intubated patients without infection (Figure 3C). Relative to the control group, multiple antibacterial immune pathways were downregulated in COVID-19 patients, with the greatest attenuation in the VAP group (Figure 3C). Upstream regulator analysis identified impaired activation of diverse cytokines in those with VAP, while IFNB1 was notably upregulated (Figure 3D). Several pro-inflammatory cytokines were downregulated in both groups compared to the controls (Figure S1). We expanded the comparison at the “early” time-point to include patients with culture-negative VAP (VAP: n=6, No-VAP: n=11) and observed similar differences at the pathway level (Figure S2).

Given prior reports demonstrating correlation between SARS-CoV-2 viral load and interferon related gene expression^14^ we next asked whether viral load differed between VAP and No-VAP patients. No differences in SARS-CoV-2 qPCR or viral reads per million (rpM) in bulk RNA-seq data were found in the days following intubation (P = 0.84 (RNA-seq), P = 0.53 (PCR), Figure S3). We also considered the possibility that differences in the number of days of steroid exposure prior to sample collection might explain results, but found no differences (P = 0.343) (Table S1).

### COVID-19 VAP is associated with impaired anti-bacterial immune signaling in monocytes, macrophages and neutrophils

**Figure 4:**
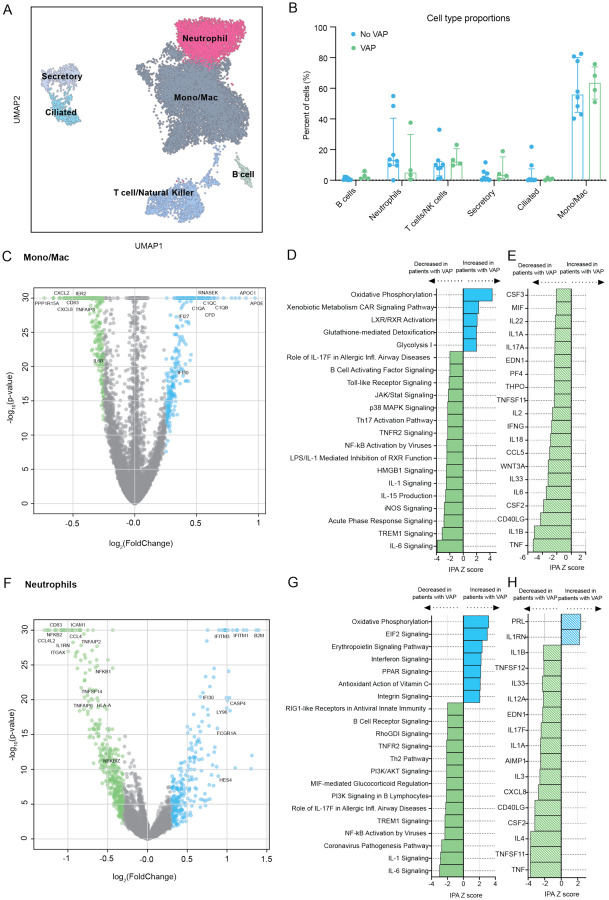
scRNA-seq demonstrates that COVID-19 VAP is associated with early impaired anti-bacterial immune signaling in lower respiratory tract monocytes, macrophages and neutrophils. **A)** UMAP of single cell RNA-seq data from patients that do or do not develop VAP at the “early” time-point, annotated by cell type. **B)** Cell type proportions in single cell RNA-seq from VAP and No-VAP patients at the “early” time-point. Bars represent the median with IQR. Statistical significance was determined by Mann-Whitney tests. None of the cell types were significantly different with a p-value <0.05. The p-values for each cell type are as follows: B cells: 0.073; Neutrophils: 0.28; T/NK cells: 0.21; Secretory: 0.46; Ciliated: 0.94, and Mono/Mac: 0.81. **C)** Volcano plot displaying the differentially expressed genes between VAP and No-VAP patients in monocytes and macrophages. **D)** Ingenuity Pathway Analysis (IPA) of key canonical pathways and upstream cytokines based on differential gene expression analysis in monocytes and macrophages of patients who develop VAP versus those who do not, with adjusted p-values < 0.05. Only significant pathways (IPA Z-score of >2 or <−2 and overlap p-value <0.05) are shown. **E)** Volcano plot displaying the differentially expressed genes between VAP and No-VAP patients in neutrophils. **F)** IPA of canonical pathways and upstream cytokines based on differential gene expression analysis in neutrophils of patients who develop VAP versus those who do not, with adjusted p-values < 0.05. Only significant pathways (IPA Z-score of >2 or <−2 and overlap p-value <0.05) are shown. All pathways and cytokines are shown in Supplementary data files 5 and 6.

To further understand the mechanism of early downregulation of key pathways involved in antibacterial responses, we next asked whether this was driven by any one local immune cell type. We performed scRNA-seq on TA specimens obtained early during disease course (median of nine days before VAP) and enriched for immune cells using CD45 selection (Methods). Clustering based upon cellular transcriptional signatures indicated that monocytes, macrophages and neutrophils were the most abundant cell types (Figure 4A, S4A) and thus we focused transcriptional assessment on these populations. A comparison of cell type proportions did not reveal statistically significant differences in populations of mono/macs, neutrophils or T cells in COVID-19 patients who subsequently developed VAP (Figure 4B).

COVID-19 patients who developed VAP had distinct cell type-specific transcriptional signatures compared to those without VAP at this “early” post-intubation time-point (Figure 4, S5, S6). With respect to mono/macs and neutrophils, we identified 532 and 693 differential expressed genes, respectively, at FDR< 0.05. Several genes with key roles in innate immunity were downregulated in both cell types in the COVID-19 patients who subsequently developed VAP versus those who did not, including *IL1Rn*, *ICAM1*, *NFKB2*, and *ITGAX* in neutrophils, as well as the neutrophil chemokines *CXCL2 and CXCL8* in mono/macs (Figure 4C, 4F, S5). In addition, similar to the bulk RNA-seq results demonstrating upregulation of type I IFN signaling at this time-point in patients who developed VAP, we noted upregulation of several interferon-induced genes including IFI27 and IFI30 in mono/macs, and IFI30, IFITM1, and IFITM3 in neutrophils (Figure 4C, F).

IPA canonical pathway analysis of gene expression within each cluster revealed downregulation of several cytokine and innate immune signaling pathways in the patients who later developed VAP at the “early” post-intubation time-point. In the mono/mac cluster, this included downregulation of IL-1, IL-6, and iNOS signaling, as well as Th17 and TNFR2 signaling (Figure 4D). Analysis of the neutrophil cluster also demonstrated attenuated IL-1, IL-6, and TNFR2 signaling and NF-κB pathways (Figure 4G). COVID-19 patients who subsequently developed VAP demonstrated upregulation of oxidative phosphorylation and glutathione detoxification in the mono/mac subset, and interferon signaling, oxidative phosphorylation and EIF2 signaling in the neutrophil cluster. Computational prediction of upstream cytokine activation by IPA revealed impaired activation of multiple pro-inflammatory cytokines in both the mono/macs and neutrophils in patients who developed VAP, including TNF, CXCL8, and IL1B, as well as downregulation of key factors important in monocyte to macrophage differentiation (*CSF2*, *CSF3*, *PF4*) (Figure 4E, H).

In the T cell population, we identified 1318 differentially expressed genes at FDR < 0.05. Genes associated with T cell recruitment, including *CXCR6, ITGA1* and *ITGA4*, which have been shown to regulate localization and retention of T cells in the lung during viral infection^15,16^, were downregulated in patients with VAP. Additionally, genes indicative of T cell activation (*CD69, CD96, LAG3, ICOS, CD27*), signaling (*CD3, ZAP70, ITK, CD8A, CD8B*), and effector functions (*IFNG, GZMA, GZMB, KLRG1*) were significantly downregulated in patients with VAP, suggesting an impairment in T cell responses (Figure S6A). IPA revealed downregulation of signaling pathways crucial for T cell recruitment, such as integrin signaling, and activation, such as CD28 signaling in helper T cells and phospholipase C signaling (Figure S6B).

### Temporal dynamics of the host response in COVID-19 patients who develop VAP

**Figure 5:**
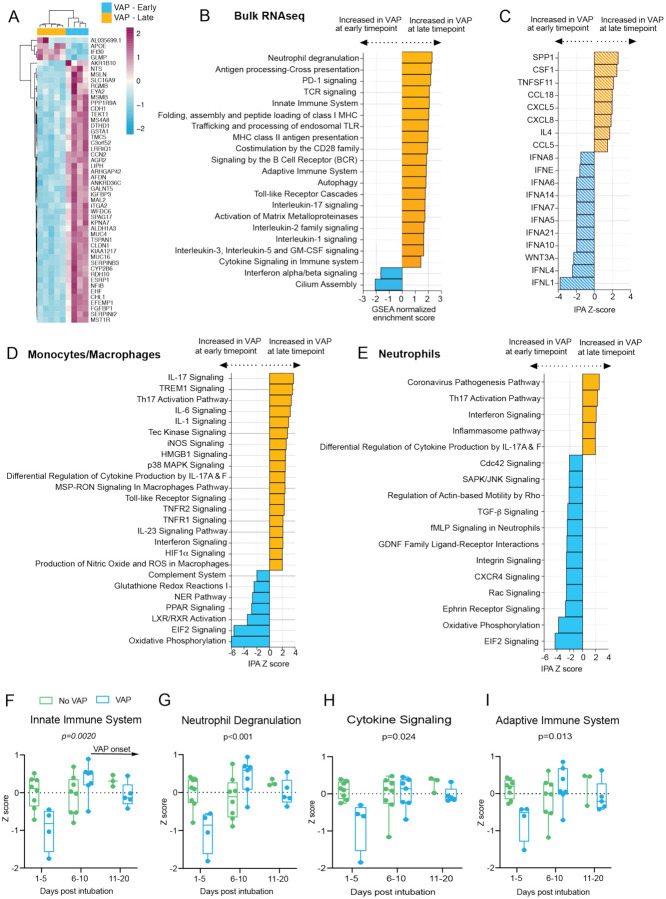
Temporal dynamics of the host response to VAP **A)** Heatmap of the top 50 differentially expressed genes by adjusted P-value between COVID-19 patients who developed VAP at the “early” time-point (blue) versus the “late” time-point (yellow) from bulk RNA-seq. **B)** Gene set enrichment analysis (GSEA) based on differential gene expression of VAP patients at the “early” vs “late” time-point from bulk RNA-seq. GSEA results were considered significant with an adjusted P-value <0.05. **C)** Ingenuity Pathway Analysis (IPA) of upstream cytokines based on differential gene expression analyses of VAP patients at the “early” vs “late” time-point from bulk RNA-seq. IPA results were considered significant with a Z-score absolute value >2 and overlap P-value <0.05. **(D-E)** Ingenuity Pathway Analysis (IPA) of key canonical pathways based on differential gene expression analysis in monocytes and macrophages (D) or neutrophils (E) from scRNA-seq of patients who develop VAP versus those who do not, with adjusted p-values < 0.05. Only significant pathways (IPA Z-score of >2 or <−2 and overlap p-value <0.05) are shown. All pathways and cytokines are shown in Supplementary data files 2, 3, 5, and 6. **(F-I)** Longitudinal analysis of selected pathway expression in VAP (blue) versus No-VAP (green) patients from bulk RNA-seq samples taken from time of intubation to onset of VAP for all patients. Pathway Z-scores were calculated by averaging Z-scores for the top 20 leading edge genes of each pathway, determined by the results of GSEA comparing VAP versus No-VAP patients at the “early” time-point. Multiple Z-scores per patient at a given time interval were averaged so that each patient corresponds to one datapoint at each interval. Samples from day 21+ after intubation are not shown due to a lack of these later time-points in the No-VAP group. VAP onset in these patients ranged from 10–39 days post intubation. Selected pathways are innate immune system (F), neutrophil degranulation (G), cytokine signaling (H), and adaptive immune system (I). Box plots represent the median and range. Statistical significance was determined by two-way ANOVA, and interaction p-values are shown.

We next investigated temporal dynamics of the lower airway host inflammatory response in COVID-19 patients from the time of intubation to development of VAP by evaluating differential gene expression between COVID-19 VAP patients at the “early” time point (median of 17 days before VAP onset, n=4) versus “late” time point (median of two days before VAP onset, n=5) by bulk RNA-seq. We identified 2705 differentially expressed genes (FDR<0.1) and unsupervised hierarchical clustering of the 50 most significant genes demonstrated clear separation of the two time-points (Figure 5A). GSEA revealed that type I interferon signaling was notably downregulated at the “late” time-point most immediately preceding VAP onset in comparison to the “early” timepoint (Figure 5B); however, expression was still significantly higher than in the No-VAP patients (Figure 2B). Several other immune signaling pathways were more highly expressed at this “late” time-point, presumably reflecting activation of an antibacterial response in the setting of bacterial pneumonia (Figure 5B). Consistent with this, upstream regulator analysis indicated increased activation of several pro-inflammatory cytokines and decreased IFNα and IFN-λ signaling at the “late” versus “early” time-points (Figure 5C).

In contrast, comparing No-VAP patients at the “early” (n=8) versus “late” (n=8) time-points yielded only two genes with a padj <0.1, both of which were interferon-stimulated genes (*RSAD2* and *CMPK2*) downregulated at the “late” time-point, suggesting that while the host response was relatively unchanged in these patients, the antiviral response attenuated over time. Indeed, GSEA revealed that type I interferon signaling, and other antiviral immune pathways were downregulated in the patients who did not develop VAP at the later time-point (Figure S7).

Next, we performed a similar comparison between the “early” and “late” time-points based on scRNA-seq data from patients who developed VAP. Differential gene expression analysis on these two populations identified 1368 differentially expressed genes (FDR<0.05) in the mono/mac cluster, and 1028 in the neutrophil cluster. IPA revealed upregulation of antibacterial signaling pathways at the later time-point, including signaling by several cytokines in the mono/mac cluster (IL-17, IL-6, IL-1, TNF, IL-23, IFN) (Figure 5D–E), congruent with the bulk RNA-seq analysis. Furthermore, we identified 1397 differentially expressed genes (FDR < 0.05) in the T cell cluster between the two time-points and noted upregulation of signaling pathways indicative of an active T cell response^17^ (e.g. ERK/MAPK, Tec kinase, and phospholipase C) in the days preceding VAP, which was also in agreement with the bulk RNA-seq results (Figure S6C).

We further assessed dynamics of host immune responses between VAP and No-VAP patients by performing longitudinal analyses of key immune signaling pathways, including all patients with available TA samples (VAP n=7, No-VAP n=10). Onset of VAP in these patients ranged from 10–39 days post intubation, with a median of 25 days, and treatment with immunosuppressants did not differ significantly between VAP and no-VAP patients (p=0.304, Fisher’s exact test). We calculated pathway Z-scores for each sample by averaging Z-scores for the top 20 leading edge genes of each pathway (Methods). Early attenuation of immune signaling in the VAP group was conspicuous, and this pattern eventually resolved later in disease course by the time secondary bacterial infection became established (Figures 5E–H). We confirmed that the observed differences between VAP and no-VAP patients were not driven by differences in treatment with immunosuppressants by comparing pathway Z-scores in patients that received immunosuppressants and those that did not at the early time-point regardless of VAP group (Figure S8).

### Lung microbiome disruption precedes VAP in COVID-19 patients

**Figure 6:**
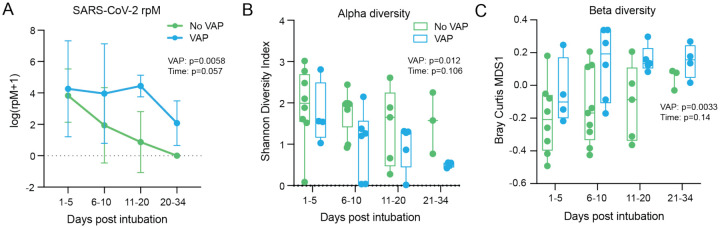
Lung microbiome community collapse precedes VAP in COVID-19 patients. **(A)** SARS-CoV-2 viral load (reads per million sequenced, rpM) over time by days since intubation in patients who develop VAP vs those who do not. For plotting purposes, log(rpM+1) was used to avoid negative values. Lung microbiome **(B)** bacterial diversity (Shannon’s Index) and **(C)** β-diversity (Bray Curtis Index, NMDS scaling) in COVID-19 patients with relation to VAP development over time by days since intubation. Box plots represent the median and range (A-C). Statistical significance was determined by two-way ANOVA. P-values <0.05 were considered significant.

We hypothesized that the innate immune suppression in patients who developed VAP would correlate with viral load. Using TA metatranscriptomics to assess the lower respiratory microbiome, we evaluated longitudinal changes in SARS-CoV-2 abundance. Although no difference was observed at the “early” timepoint (Figure S3), the trajectory of SARS-CoV-2 viral load differed significantly in patients who developed VAP (p=0.0058), although in both groups decreased over time (Figure 6A). This result suggested that COVID-19 patients who develop VAP may exhibit impaired ability to clear virus compared to those who do not, and that the lung microbiome composition may be similarly impacted.

Indeed, COVID-19 patients who developed VAP exhibited a significant reduction in bacterial diversity of their airway microbiome up to three weeks before clinical signs of infection (Shannon Diversity Index, p=0.012; Figure 6B). COVID-19 patients who developed VAP also had lower airway microbiome compositions more closely resembling each other than those from patients who did not develop VAP, across all timepoints since intubation (Bray Curtis index, p=0.0033; Figure 6C), suggesting community collapse precedes the development of VAP. All patients received antibiotics prior to collection of the first sample, suggesting that antibiotic use was not driving these differences (Table S1).

## Discussion

Secondary bacterial pneumonia contributes to significant morbidity and mortality in patients with primary viral lower respiratory tract infections^1,3^, but mechanisms governing individual susceptibility to VAP have remained unclear. Few human cohort studies have evaluated the immunologic underpinnings of VAP, and none have been reported in the context of COVID-19, which is characterized by a dysregulated host response distinct from other viral pneumonias^14,18,19^. To address this gap and probe mechanisms of VAP susceptibility in patients with COVID-19, we carried out a systems biological assessment of host and microbial dynamics of the lower respiratory tract.

Two days before VAP onset, a transcriptional signature consistent with bacterial infection was observed. This finding suggests that host response changes can occur before clinical recognition of pneumonia, highlighting the potential utility of the host transcriptome as a tool for VAP surveillance. While intriguing, this observation did not provide an explanation for differential susceptibility of some COVID-19 patients to post-viral pneumonia.

The discovery of an early suppressed antibacterial immune response in patients who later developed VAP did however, offer a potential explanation. More than two weeks before VAP onset, we observed a striking suppression of pathways related to both innate and adaptive immunity, including neutrophil degranulation, TLR signaling, complement activation, antigen presentation, and T cell receptor and B receptor signaling, as well as cytokine signaling (e.g. IL-1, IL-4, IL-12, IL-13 and IL-17). Comparison against uninfected, intubated controls confirmed the previously described paradoxical impairment in immune signaling found in patients with severe COVID-19^18^, and suggested that VAP susceptibility may be the result of disproportionate suppression of innate and adaptive pathways critical for antibacterial defense, resulting in enhanced susceptibility to opportunistic secondary infections.

Animal models of influenza have provided insight into potential mechanisms of post-viral pneumonia, although none have provided insight regarding why some individuals are more susceptible than others. In mice inoculated with influenza, for instance, virus-induced type I IFN suppresses neutrophil chemokines and impairs Th17 immunity, compromising effective clearance of bacterial infections^9,10^. Interestingly, we also observed increased type I interferon signaling in COVID-19 patients who weeks later developed VAP, and a strikingly similar impairment in Th17 signaling and other immune pathways. Desensitization to toll-like receptor (TLR) ligands after influenza infection has also been documented^20^, which is congruent with the downregulation of TLR signaling at the time of intubation observed in our bulk RNA-seq analyses.

Impaired bacterial clearance by alveolar macrophages was found to be driven by virus-related IFNγ production by T cells^21^ in a murine post-influenza model. In contrast, we found that T cells from patients who later developed VAP expressed lower levels of IFNγ at the time of intubation. This difference may relate to species-specific variations in immune signaling or intrinsic differences in the host response to influenza virus versus SARS-CoV-2^14,18^.

We asked whether certain cell types were responsible for driving the early suppression of immune signaling observed in COVID-19 patients who went on to develop VAP. No significant differences in proportions of the most abundant cell types - monocytes/macrophages, neutrophils or T cells – was observed between patients with or without VAP at the time of intubation. This finding suggests that an impairment of immune cell recruitment was not causing these differences, but rather significant gene expression differences within each of these immune cell populations.

In both the mono/mac and neutrophil populations, we observed broad downregulation of the innate immune response, and initiation of the adaptive immune response, concordant with global observations in bulk RNA-seq analyses. Further analysis revealed a downregulation of monocyte to macrophage differentiation and neutrophil chemotaxis. Further, we noted a downregulation of key pathways and transcription factors involved in antimicrobial immune responses including iNOS in mono/macs, as well as NFKB and TREM1 in mono/macs and neutrophils. Both bulk and scRNA-seq suggested an impairment in T cell recruitment, signaling, and effector functions. Overall, our data suggest that while no difference in cell type populations existed between groups, changes in the gene expression of mono/macs, neutrophils and T cells contributes to immune suppression in COVID-19 patients who later develop VAP.

SARS-CoV-2 viral load correlates with interferon stimulated gene expression^14,18^ and thus we initially hypothesized that differences in viral load between groups might relate to individual VAP susceptibility. However, we found no difference between groups at the “early” timepoint. Moreover, no differences existed in terms of immunosuppressive medication administration or clinically diagnosed immunodeficiency, suggesting that other, still unidentified mechanisms present at the time of intubation must underlie the marked suppression of immune gene expression in COVID-19 patients who went on to develop VAP.

While no difference in viral load was observed at the time of intubation, the COVID-19 patients who developed VAP exhibited impaired viral clearance over the time-course of intubation. This observation was corroborated by a prolonged antiviral type I interferon response at the “late” timepoint (median of two days before VAP onset) in patients who developed VAP versus those who did not, pointing to the persistence of suboptimal antiviral immunity in these patients. Early induction of functional SARS-CoV-2 specific T cells is associated with faster viral clearance in COVID-19 patients^22^ and likewise, we observed impairments in T cell activation and signaling in the VAP group, which further suggests a decreased ability to control the virus in these patients.

Respiratory viruses can reshape the human airway microbiome by modulating host inflammatory responses^23,24^. In mouse models of influenza, the airway microbiome exhibits expansion of several bacterial families during the course of viral infection as innate immunity is suppressed^23^. These changes increase the risk of secondary bacterial infection^23^ and have been observed in patients with chronic obstructive pulmonary disease, where suppression of the innate immune response in rhinovirus infected patients may be followed by bacterial superinfection^25,26^.

**Figure 7:**
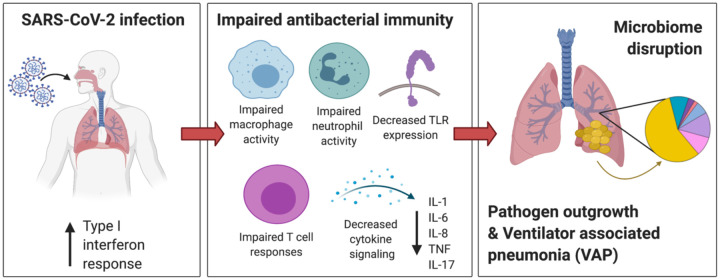
Mechanistic hypothesis of secondary bacterial pneumonia susceptibility in patients with COVID-19. Individual immune responses to SARS-CoV-2 infection drive a restructuring of the microbial community and increase susceptibility to VAP. Those predisposed to VAP have increased type I interferon responses and dysregulated antibacterial immune signaling characterized by impaired macrophage, neutrophil and T cell activity, decreased TLR signaling and impaired activation of key cytokines important for pathogen defense including IL-1, IL-6, IL-8, TNF, and IL-17. This state of suppressed immunity disrupts the lower respiratory tract microbiome, predisposing to outgrowth of bacterial pathogens and VAP.

Similarly, the innate immune suppression observed in COVID-19 patients who developed VAP was associated with airway microbiome collapse and the outgrowth of lung pathogens in advance of clinical VAP diagnosis. This finding suggests that individual immune responses to SARS-CoV-2 infection may drive a restructuring of the microbial community and increase susceptibility to VAP (Figure 7). The resulting outgrowth of a VAP-associated bacterial pathogen may elicit an antibacterial response, but the broader immunosuppressive state preceding this response may be insufficient to control the development of clinical pneumonia. Those with a lesser degree of immunosuppression may be able to respond faster and therefore control opportunistic bacterial pathogens more effectively.

These findings may also have important implications for management of patients with COVID-19 related acute respiratory failure, many of whom are now being treated with corticosteroids plus/minus IL-6 receptor blocking agents. These agents may lead to further suppression of the key pathways required for host response to secondary bacterial infection. Thus, our results emphasize the need for ongoing vigilance for VAP in patients treated with potent immunosuppressive agents, as well as the need to develop novel diagnostic and/or prognostic approaches to identifying patients at highest risk. For instance, availability of molecular biomarkers to assess a patient’s risk of VAP at the time of intubation could reduce inappropriate use of prophylactic antibiotics or immunomodulatory treatments, or signal a need for enhanced surveillance strategies. Signatures of immune dysfunction have been used as biomarkers to predict nosocomial infection in critically ill patients,^27^ although not in the context of viral infection.

Sample size is a limitation of this study; however, the reproducibility of our observations across both bulk and scRNA-seq analyses and the significant number of differentially expressed genes among the comparator groups support the validity of our conclusions. Because this study was limited to critically ill, intubated patients, we were unable to assess early stages of COVID-19, which may provide additional insight regarding determinants of secondary bacterial infection. Additionally, we were unable to assess whether epithelial cells contributed to VAP risk due to enrichment for immune cells prior to scRNA-seq. With larger cohorts, the early detection of specific immune pathway suppression and microbiome collapse could be leveraged to develop clinically useful models for identifying COVID-19 patients with increased susceptibility to secondary bacterial pneumonia.

## Materials and Methods

### Study design, cohorts, enrollment and ethics approval

We conducted a prospective case-control study of adults requiring mechanical ventilation for COVID-19 with or without secondary bacterial pneumonia. We also evaluated control patients requiring mechanical ventilation for other reasons who had no evidence of pulmonary infection (Figure 1). Patients were enrolled in either of two prospective cohort studies of critically ill patients at the University of California, San Francisco (UCSF) and Zuckerberg San Francisco General Hospital between 07/2013 and 07/2020. Both cohort studies were approved by the UCSF Institutional Review Board (IRB) under protocols 10–02701 (control patients, pre-COVID-19 pandemic) and 20–30497 (COVID-19 patients, COVID-19 Multiphenotyping for Effective Therapy (COMET) study), respectively. Of the COVID-19 patients, 19 were co-enrolled in the National Institute of Allergy and Infectious Diseases-funded Immunophenotyping Assessment in a COVID-19 Cohort (IMPACC) Network study.

For both the COVID-19 and control cohorts, if a patient met inclusion criteria, then a study coordinator or physician obtained written informed consent for enrollment from the patient or their surrogate. Patients or their surrogates were provided with detailed written and verbal information about the goals of the study, the data and specimens that would be collected, and the potential risks to the subject. Patients and their surrogates were also informed that there would be no benefit to them from being enrolled in the study and that they may withdraw informed consent at any time during the course of the study. All questions were answered, and informed consent documented by obtaining the signature of the patient or their surrogate on the consent document (or during the COVID-19 pandemic, the IRB-approved electronic equivalent, to enable touchless consent).

Many critically ill patients are unconscious at the time of intensive care unit (ICU) admission due to their underlying illness and/or are endotracheally intubated for airway management or acute respiratory failure. The patients who are not unconscious are often in pain and may have acute delirium due to critical illness and/or medications. For these reasons, many subjects are unable to provide informed consent at the time of enrollment. Because this study could not practically be done otherwise and was deemed to be minimal risk by the UCSF IRB, if a patient was unable and a surrogate was not available to provide consent, patients were enrolled with waiver of initial consent, including the collection of biological samples.

Specifically, for subjects who were unable to provide informed consent at the time of enrollment, our study team was permitted to collect biological samples as well as clinical data from the medical record obtained prior to consent. Surrogate consent was vigorously pursued for all patients; moreover, each patient was regularly examined to determine if and when s/he was able to consent for him/herself, and the nursing and ICU staff were contacted daily for information about surrogates’ availability. For patients whose surrogates provided informed consent, follow-up consent was subsequently obtained from the patient if they survived their acute illness and regained the ability to consent. For subjects who died prior to the consent being obtained, a full waiver of consent was approved by the UCSF IRB for both cohort studies. Lack of a surrogate to provide consent is common in critically ill patients. To address this, the UCSF IRB also approved a full waiver of consent for subjects in the COVID-19 cohort who remained unable to provide informed consent and had no contactable surrogate identified within 28 days. Before utilizing this waiver, we made and documented at least three separate attempts to identify and contact the patient or surrogate over a month-long period. While most patients enrolled were consented by typical processes, three died prior to consent being obtained, and five were included with a full waiver of consent due to lack of ability to consent and lack of contactable surrogate. No personally identifiable information has been included as part of this manuscript for any enrolled patients.

### Ventilator-associated pneumonia adjudication

A total of 84 adults who required intubation for severe COVID-19 (Cohort 1) and who had available TA samples were considered for inclusion in the study (Figure 1). Patients who met the Centers for Disease Control (CDC) definition for VAP^13^ with a positive bacterial sputum culture were adjudicated as having VAP for the purpose of the study (N=16); patients who did not meet these criteria, and for whom there was no sustained clinical suspicion for bacterial pneumonia during the admission, were categorized as No-VAP (N=17). VAP and No-VAP patients for whom samples at the time-points of interest were available were included in the primary analyses (VAP: N=10; No-VAP: N=13). Patients who met CDC-VAP criteria but had negative TA cultures were included in a secondary supplementary analysis only (N=5). All other patients were excluded, including patients with clinically-suspected bacterial pneumonia who did not meet CDC VAP criteria. Eight intubated patients from a recent study^18^ (Cohort 2) were included as controls and were selected because they had previously been adjudicated as having no evidence of lower respiratory tract infection. This group included four patients with the acute respiratory distress syndrome (ARDS) due to non-infectious etiologies, and four patients without ARDS who were intubated for other reasons (subdural hematoma (N=1), retroperitoneal hemorrhage (N=1), or neurosurgical procedures (N=2)).

### Tracheal aspirate sampling

Following enrollment, tracheal aspirate (TA) was collected (periodically following intubation for Study 1, or once within 3 days of intubation for Study 2), without addition of saline wash, and either a) mixed 1:1 with DNA/RNA shield (Zymo Research) for bulk RNA-seq or b) immediately processed in a biosafety level 3 laboratory (BSL3) for scRNA-seq analysis.

### Bulk RNA sequencing and host transcriptome analysis

#### RNA sequencing

To evaluate host and microbial gene expression, metatranscriptomic next generation RNA sequencing (RNA-seq) was performed on TA specimens. Following RNA extraction (Zymo Pathogen Magbead Kit) and DNase treatment, human cytosolic and mitochondrial ribosomal RNA was depleted using FastSelect (Qiagen). To control for background contamination, we included negative controls (water and HeLa cell RNA) as well as positive controls (spike-in RNA standards from the External RNA Controls Consortium (ERCC))^28^. RNA was then fragmented and underwent library preparation using the NEBNext Ultra II RNA-seq Kit (New England BioLabs). Libraries underwent 146 nucleotide paired-end Illumina sequencing on an Illumina Novaseq 6000.

#### Host differential expression

Following demultiplexing, sequencing reads were pseudo-aligned with kallisto^29^ to an index consisting of all transcripts associated with human protein coding genes (ENSEMBL v. 99), cytosolic and mitochondrial ribosomal RNA sequences and the sequences of ERCC RNA standards. Gene-level counts were generated from the transcript-level abundance estimates using the R package tximport^30^, with the scaledTPM method. Samples retained in the dataset had a total of at least 1,000,000 estimated counts associated with transcripts of protein coding genes.

Genes were retained for differential expression analysis if they had counts in at least 30% of samples. Differential expression analysis was performed using the R package DESeq2^31^. We modeled the expression of individual genes using the design formula ~VAPgroup, where VAP groups were “VAP-early”, “No VAP-early”, “VAP-late” and “No VAP-late” and used the results() function to extract a specific contrast. Separate comparisons to the control group were performed using the design formula ~COVID-19-status to compare positive and negative patients.

Significant genes were identified using a Benjamini-Hochberg false discovery rate (FDR) < 0.1. We generated heatmaps of the top 50 differentially expressed genes by FDR. For visualization, gene expression was normalized using the regularized log transformation, centered, and scaled prior to clustering. Heatmaps were generated using the *pheatmap* package. Columns were clustered using Euclidean distance and rows were clustered using Pearson correlation. Differential expression analysis results are provided in (Supplementary data file 1).

#### Pathway analysis

Gene set enrichment analyses (GSEA) were performed using the fgseaMultilevel function in the R package fgsea^32^ and REACTOME pathways^33^ with a minimum size of 10 genes and a maximum size of 1,500 genes. All genes were included in the comparison, pre-ranked by the test statistic. Significant pathways were defined as those with a Benjamini-Hochberg adjusted p-value < 0.05. Ingenuity Pathway Analysis (IPA) Canonical Pathway and Upstream Regulator Analysis^34^ was employed on genes with p<0.1 and ranked by the test statistic to identify cytokine regulators. Significant IPA results were defined as those with a Z-score absolute value greater than 2 and an overlap P value < 0.05. The gene sets in figures were selected to reduce redundancy and highlight diverse biological functions. Full GSEA and IPA results are provided in (Supplementary data files 2 and 3).

Longitudinal pathway analysis was performed using all available TA samples spanning post-intubation to VAP onset for all patients included in the bulk RNA-seq analysis. Analysis was restricted to samples with at least 1,000,000 human protein coding transcripts. Pathways of interest were selected from the significant GSEA results of the comparison of VAP vs. No-VAP patients in the “early” time-point. The top 20 leading edge genes were selected from each pathway for analysis. To calculate a Z-score for each gene, expression was normalized using the variance stabilizing transformation (VST), centered, and scaled. A pathway Z-score was calculated by averaging the 20 gene Z-scores. Multiple Z-scores per patient at a given time interval were averaged so that each patient corresponds to one datapoint at each interval. Statistical significance of pathway expression over time between VAP and No-VAP groups was calculated using a two-way analysis of variance (ANOVA) in GraphPad PRISM.

### Single cell RNA sequencing and transcriptome analysis

After collection, fresh TA was transported to a BSL-3 laboratory at ambient temperature to improve neutrophil survival. 3mL of TA was dissociated in 40mL of PBS with 50ug/mL collagenase type 4 (Worthington) and 0.56 ku/mL of Dnase I (Worthington) for 10 minutes at room temperature, followed by passage through a 70μM filter. Cells were pelleted at 350g 4C for 10 minutes, resuspended in PBS with 2mM EDTA and 0.5% BSA, and manually counted on a hemocytometer. Cells were stained with MojoSort Human CD45 and purified by the manufacturer’s protocol (Biolegend). After CD45 positive selection, cells were manually counted with trypan blue on a hemocytometer. Using a V(D)J v1.1 kit according to the manufacturer’s protocol, samples were loaded on a 10X Genomics Chip A without multiplexing, aiming to capture 10,000 cells (10X Genomics). Libraries underwent paired end 150 base pair sequencing on an Illumina NovaSeq6000.

Raw sequencing reads were aligned to GRCh38 using the STAR aligner^35^. Cell barcodes were then determined based upon UMI count distribution. Read count matrices were generated through the 10X genomics cellranger pipeline v3.0. Data was processed and analyzed using the Scanpy v1.6^36^. Cells that had <200 genes and had greater than 30,000 counts were filtered. Mitochondrial genes were removed and multi-sample integration was performed using Harmony v0.1.4^37^. Differential expression was performed using MAST v1.16.0^38^. Due to the significantly greater number of differentially expressed genes in scRNA-seq analyses, we used a more restrictive cutoff of FDR < 0.05 for significant genes. Differential expression analysis results are detailed in (Supplementary data file 4).

#### Pathway analysis

Ingenuity Pathway Analysis (IPA) Canonical Pathway and Upstream Regulator Analysis^34^ was employed on genes with p<0.05 and ranked by log2foldchange to identify canonical pathways and cytokine regulators. We utilized a more restrictive p value cutoff for scRNA-seq to ensure a similar number of genes were input into IPA. Significant IPA results were defined as those with a Z-score absolute value greater than 2 and an overlap P value < 0.05. The gene sets in figures were selected to reduce redundancy and highlight diverse biological functions. Full GSEA and IPA results are provided in (Supplementary data files 5 and 6).

### Lung microbiome analysis

RNA from tracheal aspirates was sequenced as described above. Taxonomic alignments were obtained from raw sequencing reads using the IDseq pipeline^39,40^, which performs quality filtration and removal of human reads followed by reference-based taxonomic alignment at both the nucleotide and amino acid level against sequences in the National Center for Biotechnology Information (NCBI) nucleotide (NT) and non-redundant (NR) databases, followed by assembly of reads matching each taxon detected. Taxonomic alignments underwent background correction for environmental contaminants (see below), viruses were excluded, and data was then aggregated to the genus level before calculating diversity metrics. Alpha diversity (Shannon’s Diversity Index) and beta diversity (Bray-Curtis dissimilarity) were calculated and the latter plotted using non-metric multidimensional scaling (NDMS). Comparison of alpha and beta diversity over time between VAP and No-VAP groups was calculated using a two-way analysis of variance (ANOVA) in GraphPad PRISM.

#### Identification and mitigation of environmental contaminants

To minimize inaccurate taxonomic assignments due to environmental and reagent derived contaminants, non-templated “water only” and HeLa cell RNA controls were processed with each group of samples that underwent nucleic acid extraction. These were included, as well as positive control clinical samples, with each sequencing run. Negative control samples enabled estimation of the number of background reads expected for each taxon. A previously developed negative binomial model^14^ was employed to identify taxa with NT sequencing alignments present at an abundance significantly greater compared to negative water controls. This was done by modeling the number of background reads as a negative binomial distribution, with mean and dispersion fitted on the negative controls. For each batch (sequencing run) and taxon, we estimated the mean parameter of the negative binomial by averaging the read counts across all negative controls, slightly regularizing this estimate by including the global average (across all batches) as an additional sample. We estimated a single dispersion parameter across all taxa and batches, using the functions glm.nb() and theta.md() from the R package MASS^41^. Taxa that achieved a p-value <0.01 were carried forward.

## Supplementary Material

Supplement 1

Supplement 2

Supplement 3

Supplement 4

Supplement 5

Supplement 6

Supplement 7

Supplement 8

Supplement 9

Supplement 10

Supplement 11
