# Supplementary material for: Impaired immune signaling and changes in the lung microbiome precede secondary bacterial pneumonia in COVID-19
Source: Res Sq. 2021 Apr 23:rs.3.rs-380803. Preprint. [Version 1] doi: 10.21203/rs.3.rs-380803/v1 (PMC8132240; doi:10.21203/rs.3.rs-380803/v1)
Supplement: Supplement 2 [file 37b6a8ad555d9ac3d5d94b52.pdf]

Supplementary Materials

Supplementary Table 1: Clinical and demographic characteristics of patients with COVID-19 who do or do not develop VAP at the "early" and "late" time-points, and control patients who were intubated without LRTI, included in the bulk RNA sequencing primary analysis.

	VAP (early timepoint)	No VAP (early timepoint)	P-value (early - VAP vs No VAP)	VAP (late timepoint)	No VAP (late timepoint)	P-value (late - VAP vs No VAP)	Controls (early timepoint)	P-value (+/- COVID-19)
**N**	4	8		5	8		8	
**Age in years, median (IQR)**	59.5 (56.0, 66.0)	49 (40.5, 64.0)	0.268	51.0 (48.0, 53.0)	59.5 (47.0, 64.5)	0.271	63.0 (36.0, 79.0)	0.642
**Female (%)**	1 (25.0%)	3 (37.5%)	1.00	2 (40.0%)	4 (50.0%)	1.00	4 (50.0%)	0.648
**Race (%)**								
African American	0 (0.0%)	0 (0.0%)	-	0 (0.0%)	0 (0.0%)	-	0 (0.0%)	-
Asian	0 (0.0%)	0 (0.0%)	-	0 (0.0%)	1 (12.5%)	1.00	1 (12.5%)	0.400
White	0 (0.0%)	2 (25.0%)	0.515	1 (20.0%)	2 (25.0%)	1.00	7 (87.5%)	<0.01
Other	4 (100.0%)	5 (62.5%)	0.491	4 (80.0%)	5 (62.5%)	1.00	0 (0.0%)	<0.01
Multiple	0 (0.0%)	1 (12.5%)	1.00	0 (0.0%)	0 (0.0%)	-	0 (0.0%)	1.00
**Hispanic ethnicity (%)**	3 (75.0%)	4 (50.0%)	0.576	4 (80.0%)	4 (50.0%)	0.565	1 (12.5%)	0.070
**Comorbidities (%)**								
Autoimmune	0 (0.0%)	0 (0.0%)	-	0 (0.0%)	1 (12.5%)	1.00	2 (25.0%)	0.147
Cancer	0 (0.0%)	0 (0.0%)	-	1 (20.0%)	0 (0.0%)	0.385	1 (12.5%)	0.400
COPD/Asthma	0 (0.0%)	2 (25.0%)	0.515	2 (40.0%)	1 (12.5%)	0.510	0 (0.0%)	0.495
DM	4 (100.0%)	3 (37.5%)	0.081	3 (60.0%)	4 (50.0%)	1.00	3 (37.5%)	0.650
HTN	3 (75.0%)	1 (12.5%)	0.067	2 (40.0%)	1 (12.5%)	0.510	4 (50.0%)	0.648
Obesity	4 (100.0%)	5 (62.5%)	0.491	4 (80.0%)	4 (50.0%)	0.565	3 (37.5%)	0.167
Solid organ transplant	0 (0.0%)	0 (0.0%)	-	0 (0.0%)	0 (0.0%)	-	0 (0.0%)	-
**Baseline IS meds (%)**	0 (0.0%)	0 (0.0%)	-	1 (20.0%)	1 (12.5%)	1.00	1 (12.5%)	0.400
**Treated with immunosuppressants (%)**	2 (50.0%)	3 (42.9%)*	1.00	5 (100.0%)	4 (57.1%)*	0.205	2 (25.0%)	0.633
**Steroid days before sample, median (IQR)**	1.5 (0.0, 3.5)	0.0 (0.0, 1.5)	0.343	9.0 (7.5, 16.5)	0.0 (0.0, 4.0)	**0.013**	0.0 (0.0, 0.5)	0.497
**Antibiotic use before sample (%)**	4 (100%)	8 (100%)	-	5 (100%)	8 (100%)	-	7 (87.5%)	0.400
**Length of stay, days, median (IQR)**	57.5 (44.5, 65.6)	19.0 (17.0, 34.5)	**0.017**	34.0 (28.0, 38.0)	24.0 (17.0, 34.5)	0.213	10.5 (6.0, 19.5)	<0.01
**Duration of mechanical ventilation, days, median (IQR)**	22.5 (19.0, 27.0)	14.0 (5.0, 23.5)	0.174	18.0 (7.0, 25.0)	6.5 (2.5, 16.0)	0.271	11.0 (11.0, 14.0)	0.151
**Mortality (%)**	1 (25.0%)	0 (0.0%)	0.333	2 (40.0%)	0 (0.0%)	0.128	1 (12.5%)	1.00
**Days before VAP (median [range])**	16.5 (15, 38)	-	-	2 (2, 3)	-	-	-	-
**Days post intubation (median [range])**	2 (1, 5)	2 (1, 4)	0.930	17 (8, 23)	9 (6, 19)	0.160	1 (1,3)	0.062

*Immunosuppressant use unknown for one No-VAP patient due to enrollment in a double-blinded clinical trial; N of 7 used
P-values represent comparisons of patients with or without VAP at the early or late timepoint, or all COVID-19 patients at the early timepoint compared to intubated controls. Statistical significance was determined using Fisher's exact test (categorical variables) or by Wilcoxon test

(continuous variables). Abbreviations: COPD: chronic obstructive pulmonary disease; DM: diabetes mellitus; HTN: hypertension; IQR: interquartile range; IS: immunosuppressive; VAP: ventilator-associated pneumonia.

Supplementary Table 2: Clinical and demographic characteristics of patients with COVID-19 who do or do not develop VAP at the "early" and "late" time-points that were included in the single cell RNA sequencing analysis.

	VAP (early timepoint)	No VAP (early timepoint)	P-value (early - VAP vs No VAP)	VAP (late timepoint)	No VAP (late timepoint)	P-value (late - VAP vs No VAP)
**N**	5	8		4	8	
**Age in years, median (IQR)**	53.0 (51.0, 75.0)	60.0 (47.0, 73.5)	0.883	61.5 (37.5, 74)	60.0 (47.0, 73.5)	0.865
**Female (%)**	2 (40.0%)	3 (37.5%)	1.00	0 (0.0%)	3 (37.5%)	0.491
**Race (%)**						
African American	0 (0.0%)	1 (12.5%)	1.00	0 (0.0%)	1 (12.5%)	1.00
Asian	0 (0.0%)	0 (0.0%)	-	0 (0.0%)	0 (0.0%)	-
White	0 (0.0%)	3 (37.5%)	0.231	1 (25.0%)	3 (37.5%)	1.00
Other	5 (100.0%)	4 (50.0%)	0.105	3 (75.0%)	4 (50.0%)	0.576
**Hispanic ethnicity (%)**	5 (100.0%)	4 (50.0%)	0.105	4 (100.0%)	4 (50.0%)	0.208
**Comorbidities (%)**						
Autoimmune	0 (0.0%)	0 (0.%)	-	0 (0.0%)	0 (0.0%)	-
Cancer	2 (40.0%)	0 (0.0%)	0.128	1 (25.0%)	0 (0.0%)	0.333
COPD/Asthma	1 (20.0%)	3 (37.5%)	1.00	1 (25.0%)	3 (37.5%)	1.00
DM	2 (40.0%)	4 (50.0%)	1.00	2 (50.0%)	4 (50.0%)	1.00
HTN	2 (40.0%)	3 (37.5%)	1.00	2 (50.0%)	3 (37.5%)	1.00
Obesity	2 (40.0%)	5 (62.5)	0.592	2 (50.0%)	5 (62.5%)	1.00
Solid organ transplant	0 (0.0%)	1 (12.5%)	1.00	0 (0.0%)	1 (12.5%)	1.00
**Baseline IS meds (%)**	1 (20.0%)	1 (12.5%)	1.00	0 (0.0%)	1 (12.5%)	1.00
**Treated with immunosuppressants (%)**	5 (100.0%)	3 (42.9%)*	0.081	4 (100.0%)	3 (42.9%)*	0.194
**Steroid days before sample, median (IQR)**	5 (2, 7)	0 (0,7.5)	0.327	7 (6,10)	0 (0, 8.5)	0.282
**Antibiotic use before sample (%)**	5 (100.0%)	8 (100.0%)	-	4 (100.0%)	8 (100.0%)	-
**Length of stay, days, median (IQR)**	34.0 (28, 38)	27.0 (19.5, 34.5)	0.271	38.0 (28.5, 113.5)	27.0 (19.5, 34.5)	0.174
**Duration of mechanical ventilation, days, median (IQR)**	18.0 (10.0, 22.0)	10.5 (6.5, 28.0)	0.769	23.5 (14.5, 29.5)	10.5 (6.5, 28.0)	0.443
**Mortality (%)**	3 (60.0%)	0 (0.0%)	0.035	1 (25.0%)	0 (0.0%)	0.333
**Days before VAP, median (range)**	9 (5, 16)	N/A	-	3 (2, 4)	N/A	-
**Days after intubation, median (range)**	5 (1, 10)	4 (2, 13)	0.825	8.5 (7, 18)	4 (2, 15)	0.198

*Immunosuppressant use unknown for one No-VAP patient due to enrollment in a double-blinded clinical trial; N of 7 used
P-values represent comparisons of patients with or without VAP at the early or late timepoint. Statistical significance was determined using Fisher's exact test (categorical variables) or by Wilcoxon test (continuous variables). Abbreviations: COPD: chronic obstructive pulmonary disease; DM: diabetes mellitus; HTN: hypertension; IQR: interquartile range; IS: immunosuppressive; VAP: ventilator-associated pneumonia.

Supplementary Table 3: Details of immunosuppressant use for patients included in the primary bulk and single-cell RNA sequencing analyses at both "early" and "late" time-points.

Patient ID	Analysis	Group	IS before sample	Immunosuppressant details	Steroid days
1059	Bulk RNA-seq	VAP-early	N	-	0
1154	Bulk RNA-seq	VAP-early	Y	Dexamethasone 6 mg daily	4
1196	Bulk RNA-seq	VAP-early	N	-	0
1254	Bulk RNA-seq	VAP-early	Y	Dexamethasone 6 mg daily	3
1001	Bulk RNA-seq	No-VAP-early	N	-	0
1002	Bulk RNA-seq	No-VAP-early	N	-	0
1072	Bulk RNA-seq	No-VAP-early	N	-	0
1115	Bulk RNA-seq	No-VAP-early	N	-	0
1136	Bulk RNA-seq	No-VAP-early	Y	Betamethasone 12 mg x 1	1
1158	Bulk RNA-seq	No-VAP-early	Unknown*	Baricitinib vs placebo*	0
1185	Bulk RNA-seq	No-VAP-early	Y	Dexamethasone 6 mg daily	2
1223	Bulk RNA-seq	No-VAP-early	Y	Dexamethasone 6 mg daily	2
227	Bulk RNA-seq	Control	N	-	0
413	Bulk RNA-seq	Control	N	-	0
414	Bulk RNA-seq	Control	N	-	0
462	Bulk RNA-seq	Control	N	-	0
473	Bulk RNA-seq	Control	N	-	0
477	Bulk RNA-seq	Control	Y	Dexamethasone 0.5 mg daily, fludrocortisone 0.1 mg daily	1
555	Bulk RNA-seq	Control	N		0
563	Bulk RNA-seq	Control	Y	Dexamethasone 10 mg x1, then 4 mg q6 hours	5
1047	Bulk RNA-seq	VAP-late	Y	Methylprednisolone 40 mg IV q6, tapered over 6 days to home dexamethasone 4 mg daily	23

1154	Bulk RNA-seq	VAP-late	Y	Dexamethasone 6 mg daily	7
1172	Bulk RNA-seq	VAP-late	Y	Dexamethasone 6 mg daily	8
1196	Bulk RNA-seq	VAP-late	Y	Dexamethasone 6 mg daily	10
1233	Bulk RNA-seq	VAP-late	Y	Hydrocortisone 100 mg IV q8, tapered over 1-7 days [#]; tocilizumab x1	1-7[#]
1001	Bulk RNA-seq	No-VAP-late	Y	Prednisone 50 mg x 1	1
1002	Bulk RNA-seq	No-VAP-late	N	-	0
1038	Bulk RNA-seq	No-VAP-late	Y	Tocilizumab x 2	0
1072	Bulk RNA-seq	No-VAP-late	N	-	0
1115	Bulk RNA-seq	No-VAP-late	N	-	0
1158	Bulk RNA-seq	No-VAP-late	Unknown*	Baricitinib vs placebo*	0
1185	Bulk RNA-seq	No-VAP-late	Y	Dexamethasone 6 mg daily	8
1244	Bulk RNA-seq	No-VAP-late	Y	Dexamethasone 6 mg daily	7
1047	scRNA-seq	VAP-early	Y	Methylprednisolone 40 mg IV q 6 hours, tapered over 6 days to home dexamethasone 4 mg daily	9
1154	scRNA-seq	VAP-early	Y	Dexamethasone 6 mg daily	9
1161	scRNA-seq	VAP-early	Y	Dexamethasone 10 mg x 1, then 6 mg daily; baricitinib vs placebo	4
1172	scRNA-seq	VAP-early	Y	Dexamethasone 6 mg daily	5
1357	scRNA-seq	VAP-early	Y	Dexamethasone 6 mg daily	1
1001	scRNA-seq	No-VAP-early	N	-	0
1002	scRNA-seq	No-VAP-early	N	-	0
1072	scRNA-seq	No-VAP-early	N	-	0
1115	scRNA-seq	No-VAP-early	N	-	0
1158	scRNA-seq	No-VAP-early	Unknown*	Baricitinib vs placebo*	0
1271	scRNA-seq	No-VAP-early	Y	Dexamethasone 6 mg daily; canakinumab vs placebo	10
1290	scRNA-seq	No-VAP-early	Y	Dexamethasone 6 mg daily; canakinumab vs placebo	7

1320	scRNA-seq	No-VAP-early	Y	Dexamethasone 6 mg daily x 10 days, then home prednisone 5mg daily; home tacrolimus; home mycophenolate mofetil	12
1154	scRNA-seq	VAP-late	Y	Dexamethasone 6 mg daily	7
1233	scRNA-seq	VAP-late	Y	Hydrocortisone 100 mg IV q8 hours, tapered over 1-7 days #; tocilizumab x1	1-7#
1264	scRNA-seq	VAP-late	Y	Dexamethasone 6 mg daily	10
1357	scRNA-seq	VAP-late	Y	Dexamethasone 6 mg daily	6
1001	scRNA-seq	No-VAP-late	N	-	0
1002	scRNA-seq	No-VAP-late	N	-	0
1072	scRNA-seq	No-VAP-late	N	-	0
1115	scRNA-seq	No-VAP-late	N	-	0
1158	scRNA-seq	No-VAP-late	Unknown*	Baricitinib vs placebo*	0
1271	scRNA-seq	No-VAP-late	Y	Dexamethasone 6 mg daily; canakinumab vs placebo	10
1290	scRNA-seq	No-VAP-late	Y	Dexamethasone 6 mg daily; canakinumab vs placebo	7
1320	scRNA-seq	No-VAP-late	Y	Dexamethasone 6 mg daily x 10 days, then home prednisone 5mg daily; home tacrolimus; home mycophenolate mofetil	12

*Unknown due to enrollment in double-blinded clinical trial of baricitinib vs placebo. # Exact duration unknown to due to incomplete records on transfer from an outside facility. Abbreviations: IS = immunosuppressive.

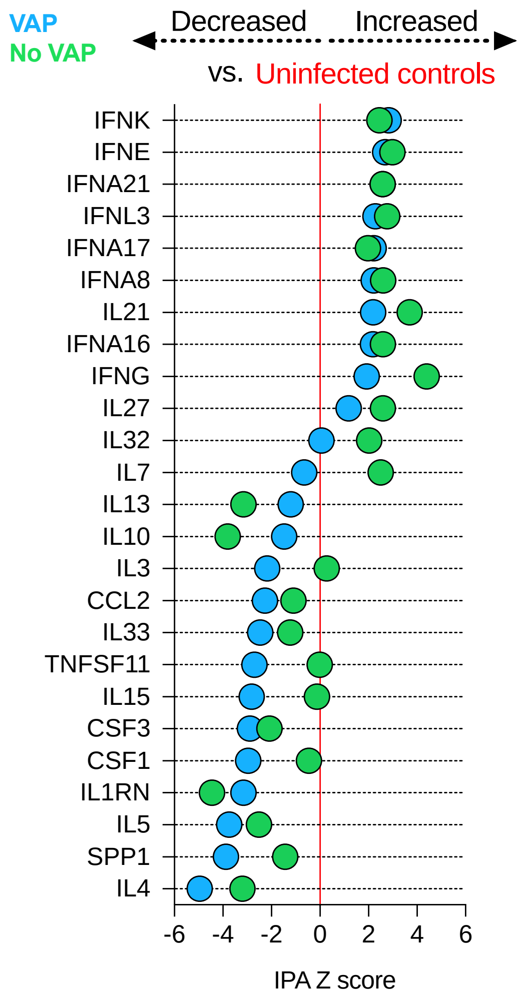

Supplementary Figure 1: Regulation of cytokines at the "early" time-point with respect to a baseline of uninfected, intubated controls. IPA upstream regulator analysis was based on differential gene expression analyses of patients with VAP vs controls and No-VAP patients vs controls. Cytokines were selected from the IPA results if they had a Z-score absolute value >2 and adjusted P-value <0.05 in both of the comparisons (VAP vs controls and No-VAP vs controls). All cytokines are shown in Supplementary data file 3.

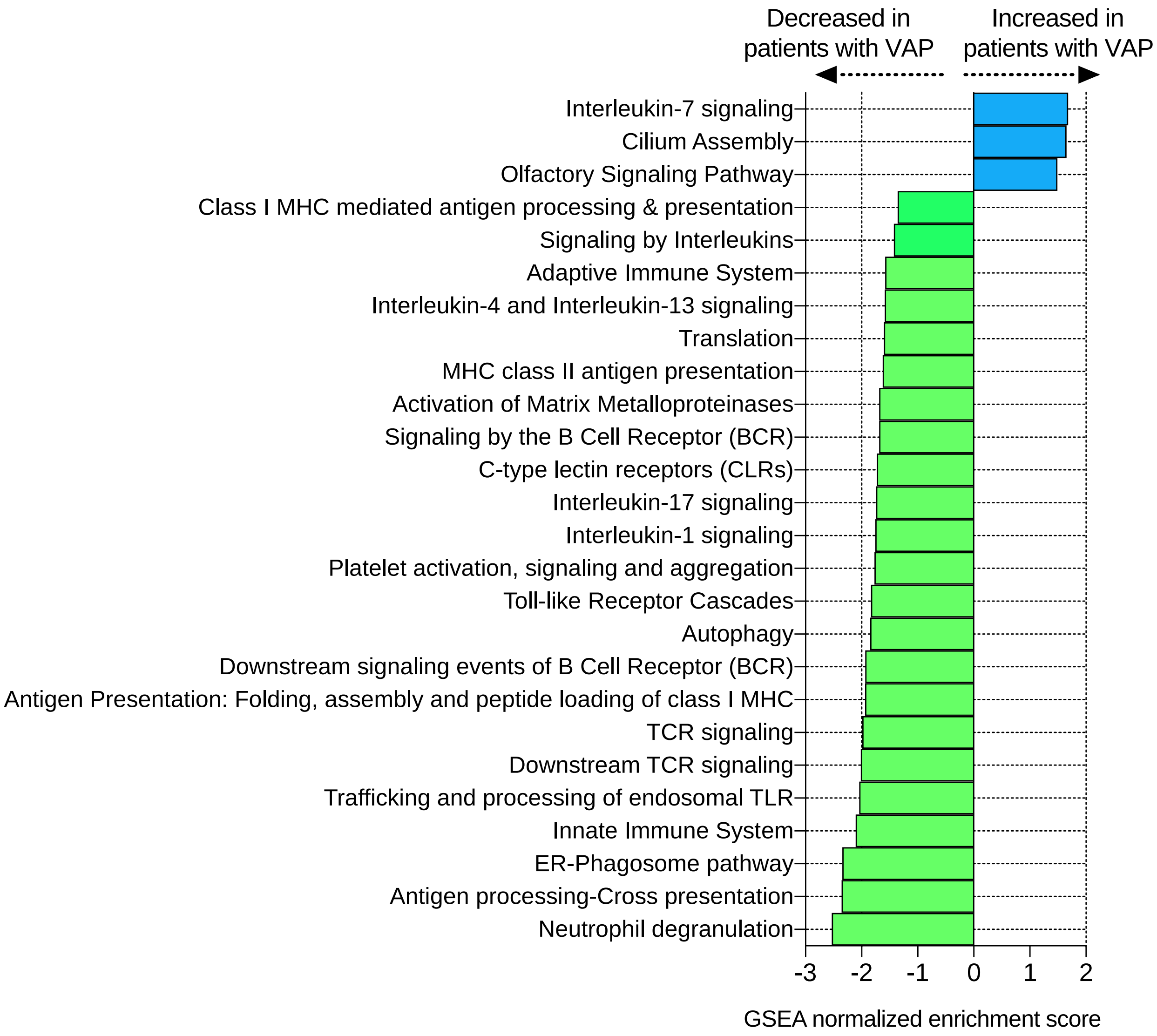

Supplementary Figure 2: Gene set enrichment analysis at the "early" time-point with an expanded definition of VAP to include culture-negative VAP cases. GSEA results with an adjusted P-value <0.05 were considered significant. All pathways are shown Supplementary data file 2.

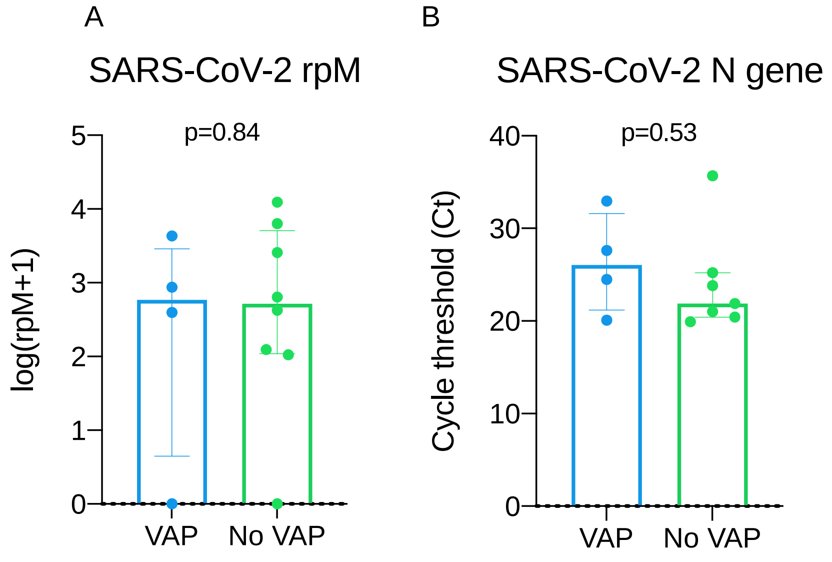

Supplementary Figure 3: SARS-CoV-2 viral load in VAP and No-VAP patients at the "early" time-point. Viral load was quantified by **A)** reads per million (rpM) from bulk RNA-seq and **B)** cycle threshold (Ct) values by qPCR for the SARS-CoV-2 N gene. The Ct value for one sample in the No VAP group was not determined. Bar plots represent the median with interquartile range. Statistical significance was determined by Mann-Whitney tests.

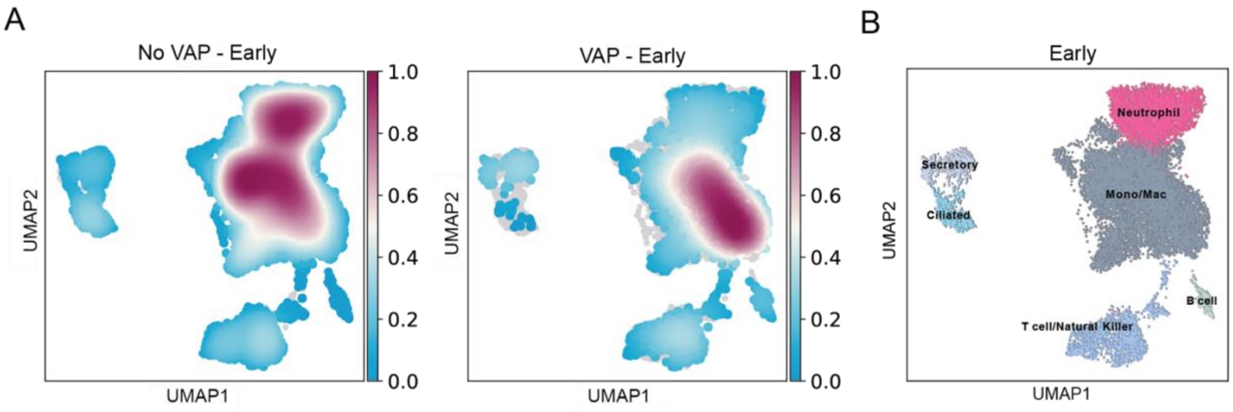

Supplementary Figure 4: (A) Density plots of single cell RNA-seq data from all VAP and No-VAP samples at the "early" time-point. **(B)** UMAP of all samples at the "early" time-point (same as Figure 3A), annotated by cell type for reference.

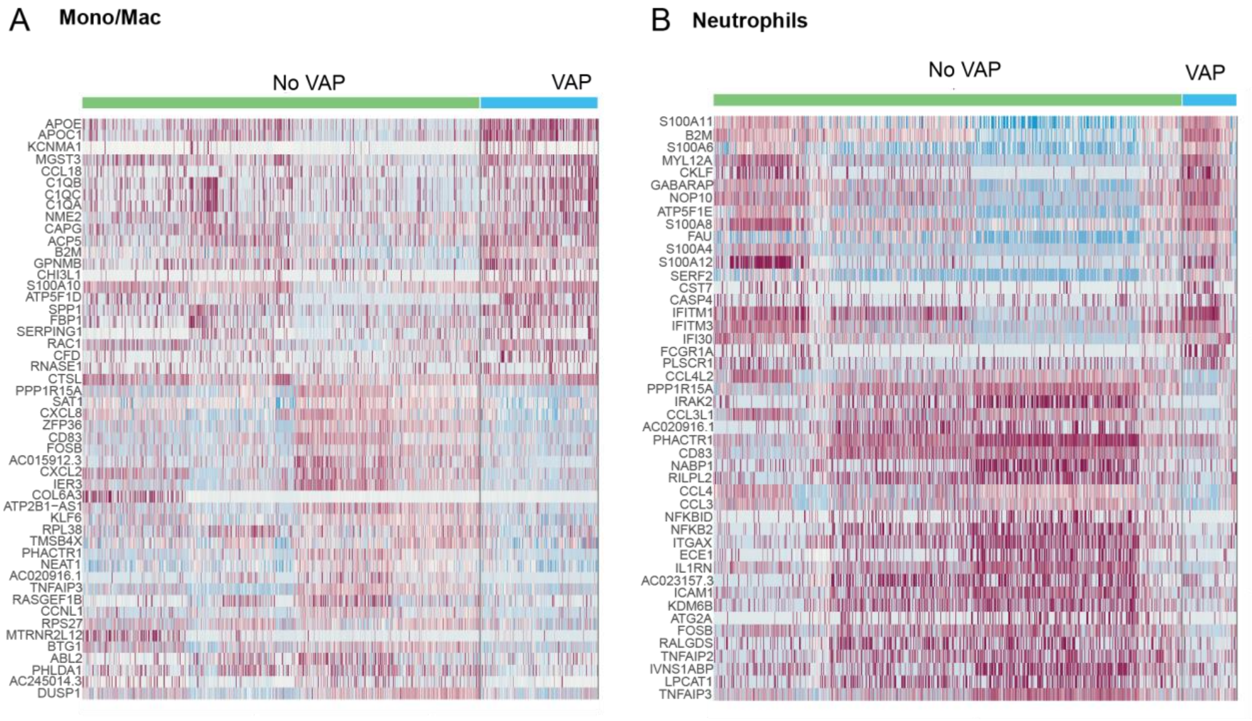

Supplementary Figure 5: Top 50 differentially expressed genes in monocytes, macrophages, and neutrophils. Heatmaps of the top 50 unsupervised differentially expressed genes between VAP and No-VAP patients in **(A)** monocytes and macrophages and **(B)** neutrophils. Differentially expressed genes are ranked based upon log2fold change +/- 0.25 and a p-value < 0.05.

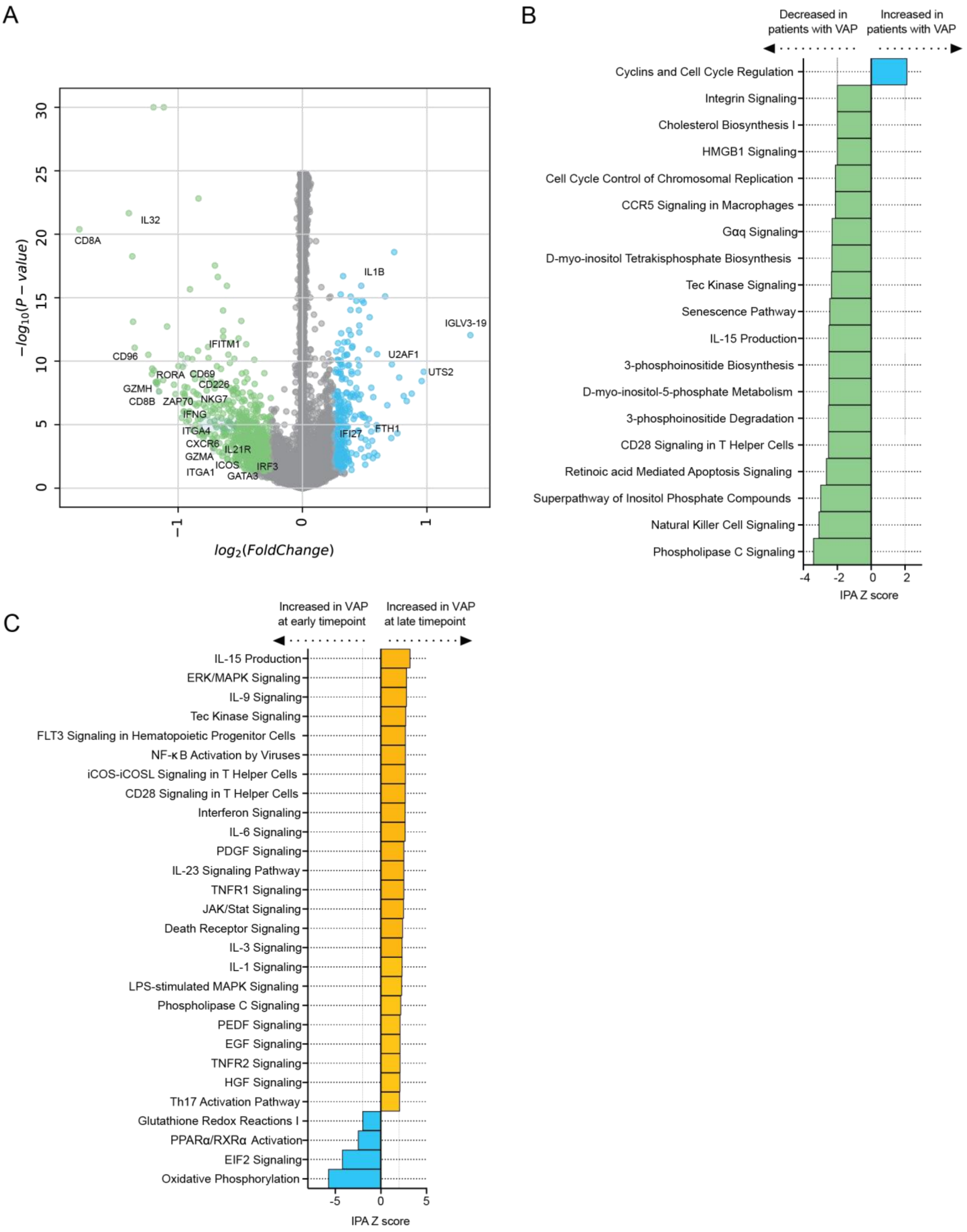

Supplementary Figure 6: (A) Volcano plot displaying the differentially expressed genes between VAP and No-VAP patients in T cells. **(B)** Ingenuity Pathway Analysis (IPA) of canonical pathways

based on differential gene expression analysis in T cells of patients who develop VAP versus those who do not, with adjusted p-values < 0.05. **(C)** IPA of canonical pathways in T cells of patients with VAP at the “early” vs “late” time-point. Only significant pathways (IPA Z-score of >2 or <-2 and overlap p-value <0.05) are shown. All pathways are shown in Supplementary data file 5.

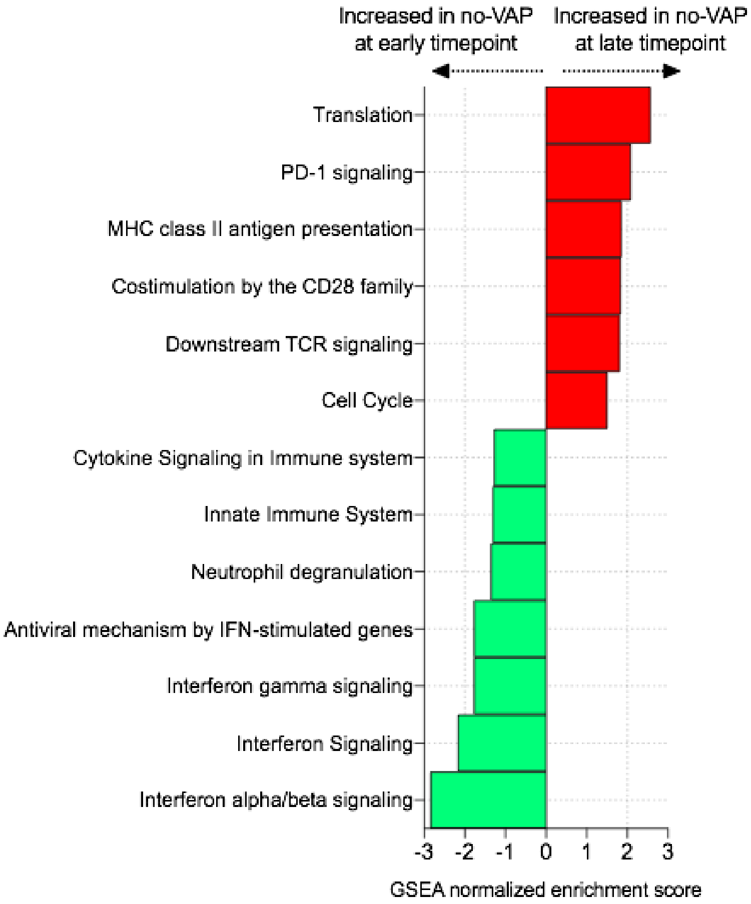

Supplementary Figure 7: Gene set enrichment analysis (GSEA) comparing patients who do not develop VAP at the “early” (green) versus “late” (red) time-points. GSEA results were considered significant with an adjusted P-value <0.05. All pathways are shown in Supplementary data file 2.

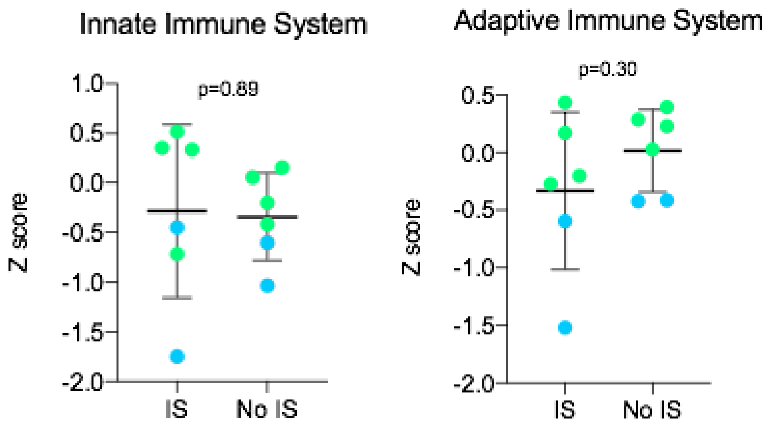

Supplementary Figure 8: Pathway expression for samples at the “early” timepoint, comparing patients who were treated with immunosuppressants before sample collection and those who were not. Pathway Z-scores for **(A)** the innate immune system pathway and **(B)** the adaptive immune system pathway were calculated by averaging Z-scores for the top 20 leading edge genes of each pathway. Samples from VAP patients are shown in blue, no-VAP patients are shown in green. IS = treated with immunosuppressants. Plots represent the mean with standard deviation. Statistical significance was determined by t-tests.

Supplementary Data Files

Data file S1: Differentially expressed genes in bulk RNA-seq analyses.

Data file S2: Gene set enrichment analysis results from bulk RNA-seq analyses.

Data file S3: Ingenuity pathway analysis for upstream regulators from bulk RNA-seq analyses.

Data file S4: Differentially expressed genes in single cell RNA-seq analyses.

Data file S5: Ingenuity pathway analysis for canonical pathways from single cell RNA-seq analyses.

Data file S6: Ingenuity pathway analysis for upstream regulators from single cell RNA-seq analyses.

Data file S7: Detailed clinical and demographic data for COVID-19 patients.

Supplementary Appendix: COMET Consortium Member list.
